# Nanotube-Assisted Motor Neuron and Neuromuscular Junction Stabilization in Spinal Muscular Atrophy: A Hypothesis for Adjunctive Therapy

**DOI:** 10.3390/neurolint18050087

**Published:** 2026-05-02

**Authors:** Almir Fajkić, Andrej Belančić, Kristina Pilipović, Valentino Rački, Silvestar Mežnarić, Tamara Janković, Elvira Meni Maria Gkrinia, Dinko Vitezić, Jasenka Mršić-Pelčić

**Affiliations:** 1 Department of Pathophysiology, Faculty of Medicine, University of Sarajevo, 71000 Sarajevo, Bosnia and Herzegovina; 2 Department of Basic and Clinical Pharmacology and Toxicology, Faculty of Medicine, University of Rijeka, 51000 Rijeka, Croatia; kristina.pilipovic@medri.uniri.hr (K.P.); silvestar.meznaric@medri.uniri.hr (S.M.); tamara.jankovic@medri.uniri.hr (T.J.); dinko.vitezic@medri.uniri.hr (D.V.); jasenka.mrsic.pelcic@medri.uniri.hr (J.M.-P.); 3 Department of Neurology, Clinical Hospital Center Rijeka, Kresimirova 42, 51000 Rijeka, Croatia; valentino.racki@medri.uniri.hr; 4 Independent Researcher, 11741 Athens, Greece; elvira.gkrinia20@alumni.imperial.ac.uk

**Keywords:** spinal muscular atrophy, neuromuscular junction, carbon nanotubes

## Abstract

Spinal muscular atrophy (SMA) therapies that restore SMN expression improve survival and motor function but often fail to fully stabilize distal motor units or sustain endurance. We propose a hypothesis-driven adjunctive approach, intended to complement SMN-restoring therapies, in which localized nanotube-enabled interfaces acting at or near the distal motor unit and neuromuscular junction enhance neuromuscular transmission reliability in surviving, remodeled motor units. The model predicts a temporal cascade: improved junctional reliability and reduced activity-dependent failure, followed by consistent motor unit output across repeated activation, and ultimately, enhanced endurance and functional reserve. Phenotype-specific responsiveness identifies patients most likely to benefit, specifically those with preserved-but-limited residual motor unit substrate accompanied by measurable neuromuscular junction instability. Drawing on shared mechanisms from ALS, spinal cord injury, and other neuromuscular disorders, we discuss mechanistic, translational, safety, regulatory, and ethical considerations. This framework links objective physiological constructs to functional outcomes, offering a mechanistically grounded path for adjunctive therapy development in SMA and related conditions.

## 1. Introduction

Spinal muscular atrophy (SMA) is a rare autosomal recessive neuromuscular disorder caused by biallelic deletions or pathogenic variants in the survival motor neuron 1 (SMN1) gene, resulting in insufficient SMN protein and progressive degeneration of lower motor neurons. Clinically, SMA is characterized by symmetric muscular weakness, hypotonia, and atrophy. Disease severity is partially modulated by the paralogous SMN2 gene, which generates limited amounts of functional SMN protein through alternative splicing, giving rise to a wide phenotypic spectrum ranging from severe prenatal or infantile-onset forms (Type 0–1) to adult-onset disease (Type 4) characterized by mild proximal weakness [1,2,3].

The introduction of disease-modifying therapies that increase SMN availability has altered the natural history of SMA, particularly when treatment is initiated pre-symptomatically [3]. These advances have reshaped SMA nosology, moving away from classifications based upon untreated disease trajectories toward frameworks based on function status, treatment response, and predicted disease course [4,5]. Nevertheless, SMN-restorative therapies do not reverse established motor neuron loss, nor do they reconstruct disrupted neuromuscular circuitry. Consequently, a substantial proportion of treated individuals continue to exhibit residual weakness, fatigability, and impaired motor endurance, highlighting an unmet need for adjunctive strategies that address downstream neurodegenerative and synaptic deficits [6,7].

In this context, neuroengineering approaches, particularly those employing nanotube-based materials, offer a compelling complement to molecular therapies (see Section 6) [8]. Applied to SMA, such platforms may reinforce compromised motor neuron–muscle connectivity, enhance neuromuscular junction function, and stabilize surviving motor networks, thereby addressing functional deficits that remain refractory to SMN augmentation alone [7,9,10,11].

Drawing on convergent evidence from amyotrophic lateral sclerosis (ALS) [12], other neurodegenerative conditions, and spinal cord injury (SCI) [13], this review proposes a hypothesis-driven adjunctive framework in which nanotube-assisted neuroengineering is leveraged to stabilize vulnerable motor neurons and neuromuscular junctions in SMN-treated SMA, with the aim of promoting motor unit stability, network resilience, and optimized long-term functional outcomes beyond SMN restoration alone. Beyond reviewing nanotube-enabled neuroengineering as a potential adjunctive strategy, the central aim of the present manuscript is to define neuromuscular junction instability as a physiologically interpretable and potentially measurable therapeutic target in treated SMA, and to organize this concept into a falsifiable translational conceptual model.

## 2. Persistent Motor Neuron and Neuromuscular Junction Dysfunction in Spinal Muscular Atrophy Despite SMN-Restoring Therapies

The advent of SMN-restoring therapies has fundamentally altered the clinical landscape of SMA, transforming a historically fatal or severely disabling disease into a chronic, treatable neuromuscular condition for many patients. Antisense oligonucleotide-based splicing correction (nusinersen), gene replacement strategies (onasemnogene abeparvovec), and orally administered splicing modifiers (risdiplam) have demonstrated efficacy in improving survival, motor milestone acquisition, and overall disease stabilization, particularly when treatment is initiated pre-symptomatically or in early infancy [12]. Nevertheless, accumulating clinical, electrophysiological, and experimental evidence indicates that SMN augmentation alone is insufficient to fully restore motor neuron integrity and neuromuscular junction (NMJ) function, especially in individuals treated after the onset of clinical symptoms [13].

Neuropathological studies and longitudinal clinical observations suggest that a critical determinant of incomplete functional recovery in treated SMA is the irreversibility of established motor neuron loss and long-standing motor unit remodeling. By the time weakness becomes clinically apparent, significant degeneration of alpha motor neurons, axonal retraction, and denervation of muscle fibers have already occurred [14]. Although SMN-restoring therapies can halt or slow further degeneration, they do not regenerate lost motor neurons nor fully reconstruct the original architecture of the motor unit [14,15]. Consequently, surviving motor neurons are often required to compensate through collateral sprouting and reinnervation, leading to enlarged but functionally vulnerable motor units that may be prone to fatigue and synaptic instability [16]. Interestingly, there is preclinical evidence that combining SMN-restoring therapies can rescue survival and motor unit function [17].

Electrophysiological studies provide evidence that NMJ dysfunction persists in SMA despite sustained SMN restoration. Repetitive nerve stimulation and single-fiber electromyography have demonstrated ongoing neuromuscular transmission defects in adolescents and adults treated with nusinersen, with significant decremental responses indicative of impaired synaptic safety margins [13]. This has been confirmed in adult patients on nusinersen therapy, where there were significantly fewer motor units in SMA patients compared to the healthy controls, even though there was some recovery of smaller motor units present [18]. Motor unit-oriented techniques such as motor unit number estimation, motor unit number index, and high-density surface electromyography, together with electrophysiological measures such as single-fiber electromyography, have increasingly been explored in SMA as tools for assessing residual motor unit substrate, transmission instability, and treatment-related physiological change, further underscoring the importance of motor unit health as an outcome domain [19]. In general, the view of SMA as a motor neuron-focused disease has shifted toward a broader view of complex interplays between glial cells, motor neurons, NMJ, and skeletal muscles, as they all play active roles in disease progression and therapeutic response [6,16,20]. At the level of the NMJ, preclinical studies have demonstrated that SMN deficiency disrupts synaptic maturation, active zone organization, and activity-dependent plasticity, leading to structurally fragile and functionally inefficient synapses [21]. While early SMN restoration can partially normalize NMJ morphology, delayed treatment appears less effective in reversing established synaptic abnormalities. Evidence from animal models suggests that postsynaptic acetylcholine receptor clustering, presynaptic vesicle release dynamics, and terminal Schwann cell function may remain altered even after SMN levels are restored, contributing to ongoing transmission instability [6,15,22,23]. These synaptic-level deficits provide a plausible mechanistic explanation for the persistence of electrophysiological abnormalities observed in treated patients.

Collectively, these findings support the concept that SMA in the treatment era should be viewed not solely as a disorder of SMN deficiency, but as a chronic motor unit disease. While SMN restoration addresses the upstream genetic cause, it does not fully resolve downstream consequences such as synaptic instability, circuit disorganization, and maladaptive compensatory remodeling. This therapeutic gap is likely to widen as treated individuals age and face increasing physiological demands on already compromised motor units. Accordingly, there is a growing recognition that optimal long-term outcomes in SMA may require adjunctive strategies aimed at stabilizing motor neurons, reinforcing NMJ integrity, and supporting activity-dependent synaptic function beyond SMN restoration alone [7,14,15].

## 3. Shared Pathobiological Features of Motor Neuron and Synaptic Degeneration Across ALS, Neurodegenerative Disorders, and Spinal Cord Injury

**Figure 1:**
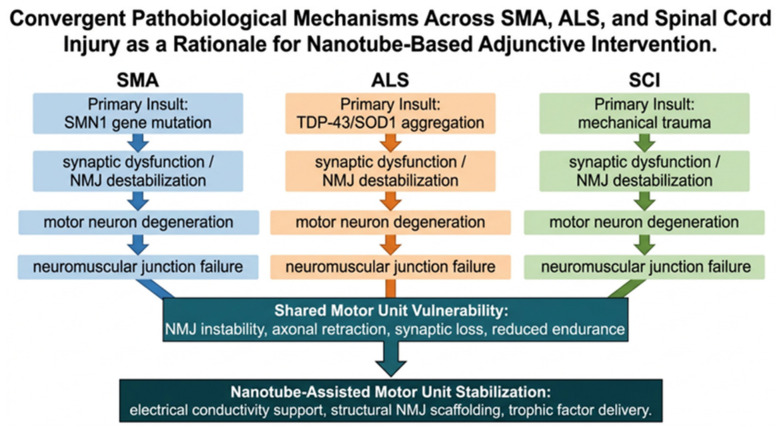
Convergent Pathobiological Mechanisms across SMA, ALS, and Spinal Cord Injury as a Rationale for Nanotube-Based Adjunctive Intervention. Three distinct disease conditions—Spinal Muscular Atrophy (SMA), Amyotrophic Lateral Sclerosis (ALS), and Spinal Cord Injury (SCI)—share a convergent cascade of motor unit pathology despite differing primary insults. In SMA, biallelic SMN1 loss drives deficient SMN protein production; in ALS, protein aggregation (TDP-43, SOD1, FUS) and RNA metabolism dysfunction trigger progressive motor neuron degeneration; in SCI, primary mechanical trauma initiates secondary excitotoxic and neuroinflammatory injury. Across all three conditions, the downstream consequences converge on early synaptic dysfunction and neuromuscular junction (NMJ) destabilization, followed by axonal retraction, motor neuron degeneration, and ultimately NMJ failure—producing a shared state of motor unit vulnerability characterized by NMJ instability, reduced synaptic safety margin, and impaired endurance. This convergence provides the mechanistic rationale for nanotube-assisted motor unit stabilization as an adjunctive intervention: carbon nanotube-based interfaces address shared downstream pathology through three complementary mechanisms—restoration of electrical conductivity at the distal motor unit, structural scaffolding of vulnerable NMJ synapses, and localized delivery of trophic factors—independent of the upstream disease-specific insult.

In SMA, motor neuron degeneration and NMJ dysfunction share remarkable pathobiological features with ALS and related neurodegenerative conditions, particularly in how synaptic structures are disrupted at the neuromuscular junction. These shared pathways provide critical mechanistic support for the adjunctive nanotube-based framework proposed for SMA in this review (Figure 1).

### 3.1. Early Synaptic Dysfunction as a Unifying Hallmark

Recent research has identified synaptic dysfunction and NMJ degeneration as early, conserved events that frequently precede the actual death of motor neuron cell bodies [24,25,26]. This “synaptopathy-first” pattern challenges traditional views that emphasized neuronal loss as the primary pathological event. In SMA mouse models, central synaptopathy at excitatory inputs onto motor neurons represents the most conserved feature of motor circuit pathology, even more consistent than changes in motor neurons themselves [24]. Similarly, in ALS, extensive proteomics studies revealed presynaptic alterations and c-Jun misactivation as convergent pathomechanisms that manifest before substantial motor neuron death [25]. The temporal sequence matters critically for therapeutic targeting. Studies demonstrate that reduced sensory synaptic excitation impairs motor neuron function via altered Kv2.1 channel dynamics in SMA, indicating that synaptic inputs actively regulate motor neuron health [27]. At the NMJ, both pre- and post-synaptic compartments show selective vulnerability, though the precise sequence and severity differs between disease contexts [26,28]. These observations suggest that preserving or restoring synaptic function could represent a disease-modifying strategy even when motor neurons are still viable.

### 3.2. Convergent Molecular Mechanisms

Multiple molecular pathways demonstrate striking convergence across motor neuron diseases. Single-cell RNA-sequencing reveals early mitochondrial dysfunction unique to motor neurons that is shared across both FUS- and TARDBP-mutant forms of ALS [29]. This mitochondrial impairment appears before other cellular changes, suggesting that it might be a fundamental vulnerability in motor neurons. Beyond mitochondria, RNA metabolism defects represent another crucial convergence point, with dysregulation of RNA processing, transport, and modification observed across familial and sporadic ALS forms [30,31]. The motor neuron m6A RNA modification repertoire specifically governs neuronal homeostasis, and FTO inhibition can mitigate ALS symptom manifestation in models [31]. Protein homeostasis defects including misfolding and aggregation of TDP-43, FUS, and SOD1 constitute well-established convergent mechanisms [30]. These protein aggregates are not just markers but actively contribute to pathology through toxic gain of function mechanisms, as demonstrated in models where mutant FUS induces selective motor neuron degeneration [32]. Impaired axonal transport further compounds these problems, since motor neurons depend critically on efficient long-distance transport of organelles, proteins, and RNA granules [33]. Recent work links KIF5A downregulation in SMA with axonal regeneration defects shared with ALS, suggesting common molecular substrates for neuronal maintenance failures [34]. Critically for SMA, the shared downregulation of KIF5A and convergent axonal transport deficits suggest that the motor neuron vulnerabilities targeted by nanotube-assisted stabilization in SMA are mechanistically grounded in evidence from these parallel disease models.

### 3.3. Selective Vulnerability Patterns

Not all motor neurons are equally affected in these diseases, and understanding selective vulnerability offers clues about protective mechanisms. Comparative studies reveal that extraocular motor neurons and slow twitch motor units often resist degeneration while limb motor neurons controlling fast twitch muscles degenerate early [26,35]. This pattern appears conserved across ALS and SMA, though the specific neuromuscular junction pathology differs [26]. In spinal and bulbar muscular atrophy, NMJ pathology correlates directly with differential motor unit vulnerability, indicating that synaptic alterations are central to determining which motor neurons survive [36]. Several factors appear to confer resistance. Synaptotagmin 13 has emerged as a neuroprotective factor across motor neuron diseases, with higher expression correlating with reduced vulnerability [37]. The intrinsic properties of the motor neurons themselves also matter, as differences in metabolic demands, calcium buffering capacity, and synaptic input patterns all contribute to vulnerability versus resistance [35]. Interestingly, opposite synaptic alterations occur at different motor unit types in the same ALS model, suggesting that motor unit identity fundamentally shapes disease progression [38]. In SMA specifically, understanding which motor unit populations retain sufficient integrity to benefit from nanotube-assisted stabilization is central to the phenotype-specific responsiveness model proposed in this review.

### 3.4. Spinal Cord Injury as a Comparative Model

SCI provides both overlapping and distinct insights into motor neuron and synaptic degeneration mechanisms. Following SCI, motor neurons below the injury level undergo progressive changes including alterations in excitability, synaptic remodeling, and eventual atrophy, though frank motor neuron death is less prominent than in ALS or SMA [39,40]. The NMJ becomes disrupted following SCI through both anterograde effects (loss of descending input) and retrograde signaling from denervated muscle [40]. Importantly, SCI triggers different glial responses and neural circuit remodeling compared to peripheral nerve injury, despite both affecting the neuromuscular system [39]. Translatomic analysis comparing regenerating motor neurons after sciatic nerve injury with degenerating motor neurons in ALS revealed both shared and divergent molecular signatures [41]. This suggests that motor neurons retain intrinsic regenerative capacity that is somehow blocked or overwhelmed in neurodegenerative contexts. Understanding why SCI motor neurons can sometimes recover function, while ALS motor neurons might reveal critical therapeutic targets related to the cellular environment or cell-autonomous factors. These observations are directly relevant to SMA: similar to post-SCI motor neurons, surviving motor neurons in treated SMA patients undergo progressive synaptic remodeling and loss of trophic support, reinforcing the rationale for nanotube-based structural stabilization as an adjunctive strategy.

### 3.5. Neuromuscular Junction Pathology Across Diseases and Implications for Therapeutic Strategies

The NMJ represents a critical nexus where pathology manifests across these conditions, yet the specific patterns vary. In SMA, pre-synaptic motor nerve terminals show defects including swelling and retraction, while post-synaptic acetylcholine receptor clusters fragment and lose apposition with nerve terminals [20,21,28]. P53 reduction can ameliorate NMJ loss without affecting motor neuron pathology directly, indicating partially independent mechanisms [42]. In ALS, integrative proteomics highlights presynaptic alterations as primary events, with synaptic vesicle dynamics and neurotransmitter release machinery particularly affected [25,43]. Cell to cell communication at the NMJ becomes disrupted in motor neuron disease through multiple mechanisms: loss of trophic support from muscle and perisynaptic Schwann cells, altered neurotransmission, and inflammatory changes in the synaptic microenvironment [44]. These changes create a deteriorating cycle where motor neuron dysfunction leads to NMJ pathology, which in turn worsens motor neuron health. Recent organ-on-chip models incorporating motor neurons with blood–brain like barriers allow for the investigation of how systemic factors and local synaptic changes interact [45].

The convergence of pathobiological features across ALS, SMA, other neurodegenerative disorders, and aspects of SCI has important therapeutic implications. Targeting early synaptic dysfunction before motor neuron death occurs could provide a wider therapeutic window. Addressing shared molecular mechanisms like mitochondrial dysfunction, RNA dysregulation, or impaired axonal transport might benefit patients across diagnostic categories. The selective vulnerability patterns suggest that understanding and enhancing protective factors such as Synaptotagmin 13 could stabilize vulnerable motor neuron populations [37]. Nanotechnology-based interventions including carbon nanotube scaffolds are being explored for their potential to stabilize motor neurons and NMJs while promoting axonal regeneration [46,47]. In cervical SCI models, early nanoparticle intervention preserves motor function and improves innervation, while combinatorial approaches using nanoparticles with a microtubule stabilizer such as epothilone D enhance regeneration even in chronic injury [46]. These scaffolds can modulate the injury microenvironment, provide structural support for regenerating axons, and deliver therapeutic molecules to targeted regions. For SMA specifically, where SMN-restoration therapies have shown substantial benefit but do not fully prevent disease progression in all patients, nanotube-assisted stabilization of motor neurons and NMJs could offer adjunctive therapeutic value [20]. The rationale is that even when SMN levels are partially restored, motor neurons and their synapses might benefit from additional structural and trophic support, particularly during critical developmental windows or in patients with more severe disease. Integration of nanotechnology with gene-targeted therapies remains largely unexplored but represents a promising translational direction based on preclinical evidence from related disease contexts [46,47].

Despite progress, key gaps remain in understanding when synapses vs. motor neurons degenerate across disease stages and patient groups. The molecular basis of selective vulnerability is still unclear, limiting the prediction of which motor neurons will fail and which protective pathways could be boosted therapeutically [48]. Also unresolved is how motor neurons respond differently in neurodegeneration vs. injury, which matters for designing regenerative strategies [49]. Translating nanotechnology from preclinical models to humans remains challenging due to biocompatibility, effective delivery to motor regions, optimal intervention timing, and integration with existing therapies [50]. Cross-disease studies using human iPSC-derived motor neurons, organ-on-chip platforms, and advanced patient imaging could help close these gaps, alongside standardized outcomes that capture synaptic function and NMJ integrity, not only motor neuron survival. Table 1 compares key features of SMA, ALS, and spinal cord injury relevant to nanotube-assisted motor unit stabilization, highlighting their shared mechanisms.

## 4. Cross-Disease Evidence Supporting Structural and Functional Stabilization of Vulnerable Motor Neurons: Relevance for SMA

In SMA, residual motor neuron dysfunction persists despite SMN restoration, driven by altered intrinsic excitability, synaptic and dendritic structural loss, extracellular matrix disruption, impaired glial support, and deficient growth factor signaling. Evidence from ALS and related neurodegenerative conditions—where analogous mechanisms have been more extensively characterized—provides a mechanistic framework that directly informs adjunctive stabilization strategies for SMA.

Animal studies indicate that in SMA, motoneurons show hyperexcitability, characterized by a hyperpolarized threshold for action potential generation, prior to motoneuron loss [51]. A similar disturbance of nerve excitability occurs in ALS, where motor neuron activity becomes progressively dysregulated. In early disease stages, this dysregulation appears presented as cortical and axonal hyperexcitability [52,53], resulting from abnormal sodium conductance clinically presenting in the form of fasciculations. As the disease advances, this state of hyperexcitability transitions to neuronal hypoexcitability [54]. Therapy strategies in ALS aim to counteract these excitability disturbances. Early treatment with riluzole stabilizes voltage-gated sodium channels, inhibits glutamate release, and interferes with intracellular signaling cascades triggered by the activation of excitatory amino acid receptors [55], thereby supporting functional stabilization by transiently increasing intracortical inhibition [52]. Edaravone, used as an adjunct therapy to riluzole, is a potent free radical scavenger approved for early-stage ALS to slow disease progression [56]. Additionally, the antiarrhythmic agent mexiletine, a sodium channel blocker, reduces motor evoked potential and partially stabilizes motor neuron firing properties [53]. Arimoclomol, a co-inducer of the heat shock proteins (HSPs), has demonstrated neuroprotective effects in neurodegenerative models including ALS. Similarly, the natural compound celastrol induces HSPs and exerts neuroprotection in ALS and SCI models through anti-inflammatory mechanisms, justifying further investigation into its effects on glial cells [57]. While these ALS pharmacological strategies directly target excitability dysregulation, analogous sodium channel dysfunction and hyperexcitability have been described in SMA motor neurons [51], suggesting that nanotube-assisted stabilization of neuronal excitability may offer complementary benefit in the SMA context without pharmacological intervention.

The axon initial segment (AIS), the site of proximal axon action potential initiation, contains a high density of ion channels and organized by ankyrin-G–based cytoskeletal scaffolding. Disruption of the AIS structure has been implicated in several neurological conditions, including Alzheimer’s disease (AD), as well as following traumatic and ischemic brain injuries [58]. Mutations in AIS ion channel subtypes are known to cause epilepsy, pointing out the importance of AIS in neuronal excitability. Although direct evidence of AIS alterations in SMA is lacking, the disease is associated with axonopathy [59] and altered neuronal excitability due to sodium channel dysfunction [60], which may indirectly suggest AIS involvement. Maintenance or reassembly of AIS should be considered an important component in regenerative strategies. Experimental studies further support the role of AIS dysfunction in MN disease. In TDP-43 C9ORF72 hiPSC MN and ALS patient-derived MN, AIS elongation is associated with impaired plasticity, intrinsic hyperexcitability, and may drive spontaneous myofiber contractions. Notably, alternations in AIS function appear to precede the onset of TDP-43 pathology and are considered as the early changes in MN vulnerability that may initiate MN pathology [54]. Furthermore, TDP-43 and C9ORF72 interact with SMN and are involved in stress granule assembly [61], suggesting converging molecular pathways across MN disorders. AIS alternations have also been shown experimentally in AD models and AD patients [62,63]. Benitez et al. reported that astrocytic disfunction, evidenced by transcriptomic and proteomic alterations in APP/PS1 astrocytes, are linked to AIS abnormalities. Normalization of AIS parameters toward control levels can be achieved experimentally by inhibiting retinoic acid degradation, blocking P2X7 receptor activity, and supplementing with the ADNP-derived peptide NAP [63]. Although direct AIS evidence in SMA remains limited, the well-documented axonopathy and sodium channel dysfunction in SMA motor neurons [59,60]—paralleled by AIS alterations in TDP-43/C9ORF72 models—support AIS stabilization as a mechanistically relevant target for nanotube-assisted intervention in SMA.

Synaptic loss [64] and NMJ instability [21] are hallmark features of SMA pathology [65]. SMN deficiency impairs axonal transport, resulting in neurofilament accumulation at the NMJ, which disrupts NMJ maturation and produces smaller, less perforated endplates with partial denervation and compromised synaptic function. These presynaptic alterations ultimately induce retrograde damage to motor neurons [7,21]. Supporting the importance of presynaptic integrity, Moradi et al. [66] demonstrated that restoring Munc13-1, a key component of the tripartite Rab3A/α-RIM/Munc13-1 complex that regulates synaptic vesicle docking and priming, ameliorates presynaptic NMJ defects in SMA models by enhancing synaptic vesicle fusion and neurotransmission.

More broadly, synaptic dysfunction in SMA precedes motor neuron death and involves glial-mediated disruptions at both the central synapses and NMJs [6]. A similar sequence occurs in ALS, where neuromuscular synapse degeneration also precedes motor neuron loss [67], suggesting that clinical interventions aimed at preserving these synapses could assist in maintaining neuromuscular communication in both disorders. Supporting that concept, Cantor et al. [68] preserved the function of neuromuscular synapses in SOD1G93A mice after disease onset by applying an agonist antibody to MuSK, a receptor tyrosine kinase essential for neuromuscular junction formation and maintenance. This intervention slowed the loss of neuromuscular connections, supported motor neuron integrity, enhanced motor output, and prolonged the survival of SOD1G93A mice. Mora et al. [69] demonstrated that overexpression of extended synaptotagmin 1 (Esyt1), an endoplasmic reticulum-tethered protein that acts as a pivotal mediator of cytosolic protein secretion [70], that is, downregulated in early ALS, in V1 interneurons increased the number of inhibitory synapses on motor neurons, prevented motor neuron loss, and improved motoric features of SOD1G93A mice. These findings highlight inhibitory synapses as key modulators of the complex interplay between spinal V1 inhibitory interneurons and motor neurons. Further emphasizing early synaptic vulnerability, in SMNΔ7 SMA mice, motor neuron dendrites actively phagocytose degenerating presynaptic terminals with microglial assistance. This results in early embryonic loss of glutamatergic synapses, reduced neuronal excitability, and elevated susceptibility to degeneration [71].

Studies have shown that ECM alterations, such as reduced laminin alpha 2 (LAMA2) expression, occur in the spinal cords and peripheral nerves of SMA mice [72,73]. Concurrently, SMN deficiency in Schwann cells impairs myelination, neuron–glial interactions, and axon stability [74]. Restoration of SMN expression in Schwann cells corrects ECM defects and improves NMJ pathology but does not prevent motor neuron soma loss [74]. Non-neuronal mechanisms clearly play an important role in SMA pathology [6]. Extensive understanding of their synergistic interactions is necessary to reveal their interplay in disease progression. Furthermore, clarifying how these mechanisms drive neurodegeneration could facilitate the development of complementary therapies and a more holistic SMA treatment strategy. Glial cells are key regulators of ECM, modulating phagocytosis and synapse stability in SMA [6,75], which further spotlights the importance of the glial contributions in the disease development.

Neurotrophic factors represent an additional significant interest because they promote neuronal survival, so their signaling pathways are considered as promising treatment targets for stabilizing vulnerable motor neurons in SMA. However, despite extensive preclinical investigations of neurotrophic factors such as BDNF, CNTF, GDNF, IGF-1, VEGF, FGF, HGF, BMP-7, and G-CSF, limited efficacy has been observed in ALS patients. This limited success likely demonstrates the complex and overlapping nature of their signaling pathways, which complicates effective treatment [57,76].

## 5. Spinal Cord Injury as a Translational Model for Nanotube-Enabled Neuroregeneration and Circuit Support

### 5.1. Introduction

SCI serves as a translational model for nanotube-enabled neuroregeneration due to its combination of axonal disconnection, NMJ instability, glial scarring, and measurable functional deficits—pathobiological features that closely parallel the motor unit vulnerability in treated SMA described in Section 2, Section 3 and Section 4. Unlike SMA and ALS, SCI offers defined anatomical lesions and standardized outcome measures, making it a rigorous in vivo platform for CNT-based circuit stabilization strategies [77,78,79].

### 5.2. Pathophysiological Parallels Between SCI and Motor Neuron Disorders

Although traumatic in origin, SCI reproduces several biological features characteristic of motor neuron diseases. Following the primary mechanical insult, a cascade of secondary injury processes unfolds [80], with surviving motor neurons below or near the lesion often undergoing gradual “dying-back” degeneration, characterized by distal axon retraction, loss of synaptic inputs, and reduced trophic support—mechanisms also central to SMA and ALS pathology [81,82,83]. Thus, SCI serves as an accelerated and spatially defined model of circuit breakdown. Interventions that restore connectivity or stabilize synapses in SCI may have conceptual and mechanistic relevance for chronic motor neuron degeneration.

### 5.3. Mechanisms of Nanotube-Enabled Neuroregeneration

Nanotubes, particularly CNTs, possess structural and physicochemical features such as nanoscale architecture, electrical conductivity, mechanical resilience, surface functionalization, and antimicrobial activity that closely match the requirements for neural repair [84]. CNT diameters correspond closely to the approximate dimensions of extracellular matrix fibrils and neurites, enabling intimate interactions with neuronal membranes and growth cones [85]. Tissue damage is often associated with varying levels of infection, and the presence of certain implanted materials can further impede the body’s natural healing process. To address this, researchers have CNTs with antimicrobial properties as a way to better manage infections in damaged tissue. Recent studies indicate that the primary antimicrobial mechanism of multi-walled CNTs is physical rather than chemical. These nanotubes can directly interact with microbes, causing structural disruption of their cell walls and membranes, which ultimately leads to cell damage and death [84,86]. Together, these properties make nanotubes uniquely capable of serving as both regenerative scaffolds and bioelectrical interfaces.

Aligned CNT arrays provide topographical cues that guide axonal growth across lesion gaps. Functionalized MWCNTs (f-MWCNTs) have been shown to deliver therapeutics like BDNF while downregulating inhibitors such as Nogo receptor (NgR) and RhoA, enhancing neuroprotection in vitro and in vivo [87]. CNT-based composites have also been explored as carriers for enzymes such as chondroitinase ABC, which degrades inhibitory chondroitin sulfate proteoglycans (CSPGs) within the glial scar, thereby improving regenerative permissiveness [77,88]. Moreover, in vitro and in vivo studies show increased synaptic density, enhanced maturation of synaptic contacts, and elevated miniature excitatory postsynaptic currents when neurons interface with CNT networks. These effects suggest that nanotubes may promote the formation of relay circuits and compensate for lost long-distance projections through local network reorganization [77]. In addition, CNTs can serve as conductive interfaces that modulate neuronal excitability. Electrical stimulation via CNT-based electrodes has been linked to enhanced calcium signaling, activation of various transcriptional pathways, and facilitation of long-term potentiation in spared circuits. This bioelectrical support may help maintain functional activity in partially disconnected motor pathways [9,89].

### 5.4. Preclinical Evidence from SCI Models

Rodent SCI models provide quantitative platforms for evaluating CNT-based therapies at anatomical, electrophysiological, and behavioral levels [90]. Functionalized single-walled carbon nanotubes (SWNT-PEG) administered into the lesion epicenter in T9 transection rat models reduced lesion cavity volume, increased neurofilament-positive fibers and corticospinal tract axon presence within the injured site, and led to modest improvements in hindlimb locomotor function without exacerbating reactive gliosis or hyperalgesia, as assessed by BBB locomotor scores and gait analysis [91]. Similarly, CNT/Nafion nanocomposites injected after SCI showed reduced lesion volume, increased axonal representation, and modest hindlimb functional recovery in open-field and rotarod tests, indicating the promotion of axonal repair and regeneration in rats [92]. Importantly, these benefits are not limited to acute injury. Some in vivo and in vitro studies indicate that CNT implantation in chronic SCI still supports plasticity and circuit remodeling, expanding the potential therapeutic window [87,93]. Compared with stem cell transplantation, which carries significant risks such as tumorigenicity, ectopic engraftment, and immune incompatibility in preclinical models of SCI, CNTs offer a biocompatible, non-cellular platform that supports tissue ingrowth and structural guidance without introducing proliferative cells that may form teratomas or elicit host rejection. By providing a permissive extracellular matrix-like environment and facilitating the targeted delivery of trophic factors or endogenous cells, CNTs serve as complementary or synergistic components in combinatorial regenerative strategies, enhancing safety and therapeutic potential [94,95]. Various in vitro and in vivo SCI models enable longitudinal study of how nanomaterials interact with glial and immune cells, a crucial step for predicting long-term safety and compatibility in the CNS. The post-injury SCI microenvironment is characterized by reactive astrocytes, activated microglia, and infiltrating immune cells. While initially protective, this response becomes chronically inhibitory. Functionalized CNTs, such as polyethylene glycol (PEG) functionalized SWCNTs (PEG-SWCNTs), offer opportunities to reshape this environment by reducing astrogliosis in vivo. Using astrocytes from GFAP-null mice, the changes associated with PEG-SWCNTs were shown to depend on astrocyte GFAP expression [96].

### 5.5. Translational Considerations and Future Directions

SCI offers strong translational alignment with human clinical assessment tools, including electrophysiology, imaging, and standardized functional scales. Demonstrating that CNT-based therapies improve conduction, coordinated motor output, and durable synaptic connectivity in SCI models strengthens the rationale for broader neurological applications. Key translational issues include the biocompatibility and long-term persistence of CNTs, mechanical matching to avoid secondary tissue damage, controlled electrical properties to prevent aberrant hyperexcitability, and scalable, standardized manufacturing for clinical use [97,98,99]. Ongoing large-animal studies and advances in nanotube purification and functionalization are helping to address these barriers. The integration of CNT scaffolds with neural stem cell transplantation, growth factor delivery, optogenetics, and closed-loop stimulation systems represents a promising frontier. SCI provides a structured platform for testing such multimodal strategies, with lessons directly transferable to other CNS injuries such as stroke or optic nerve degeneration [100,101,102].

In conclusion, SCI serves as a valuable translational model for developing nanotube-enabled neuroregenerative therapies. Its combination of axonal disconnection, synaptic instability, inflammatory barriers, and measurable functional deficits reflects central challenges across motor system disorders. By enabling the precise evaluation of how conductive, structurally aligned, and biofunctionalized nanotube systems influence axonal regrowth, synapse formation, and circuit reorganization, SCI research establishes the mechanistic and safety foundation for a new generation of nanotechnology-driven neural repair strategies.

## 6. Mechanistic Interactions Between Nanotubes, Motor Neurons, and Neuromuscular Junctions

Nanomaterials have the potential to be applied in regenerative medicine, pharmacology and stem cell research and revolutionize the future of medicine. They have been the focus of recent studies regarding their capability for the prevention, diagnosis, and treatment of various CNS disorders [103]. Among nanomaterials, one of the most commonly used and studied are CNTs [104], which have a carbon-based structure, and thus show much lower toxic potential compared to other types of nanoparticles [104]. Carbon nanomaterials show great promise for use in nervous system pathology through different modes of biomedical applications [91].

### 6.1. Surface Functionalization Strategies for Enhanced Neuromuscular Integration

In a clinical context of conditions such as SMA or ALS, the nanotubes’ natural hydrophobicity and potential toxicity must be addressed [105]. This is achieved through surface functionalization techniques, which can broadly be categorized into covalent and non-covalent methods [106]. In the latter, nanomaterials are “wrapped” around by aromatic molecules, bio-polymers, or coated with proteins such as laminin (major component of the extracellular matrix in the NMJ) [107]. This approach is often preferred for neuroregeneration because it preserves the pristine structure of the nanotube, maintaining its superior electrical conductivity, which is critical for motor neuron signaling. The former involves creating permanent chemical bonds on the nanotubes’ surface, and while it can slightly reduce conductivity, it offers much a higher stability for long-term implants [108]. A recent innovation uses polydopamine, a material that allows for the easy and stable attachment of almost any biological cue to the nanotubes’ surface, creating a highly biomimetic interface for the NMJ [109].

### 6.2. Electrical, Structural, and Biochemical Modulation of the Neuromuscular Junction by Carbon Nanotubes

The therapeutic potential of CNT-assisted strategies in SMA is hypothesized to rely on three complementary mechanisms: enhancement of electrical conductivity, provision of structural scaffolding, and modulation of the synaptic microenvironment. Together, these mechanisms aim to stabilize and reinforce the NMJ, a critical site of pathology in SMA [89,92,110].

In SMA, progressive axonal degeneration compromises this process, resulting in weakened or intermittent signal transmission and subsequent muscle atrophy. Due to their exceptional electrical conductivity and high aspect ratio, CNTs may facilitate improved charge transfer when interfaced with neuronal membranes [86,110,111]. By reducing local electrical resistance, CNTs could support more reliable action potential propagation in compromised axons. Furthermore, CNTs have been proposed to function as conductive bridges across sites of structural or functional discontinuity [112,113,114]. In this context, they may act as bypass conduits between degenerating axons and muscle fibers, helping preserve effective excitation and maintaining muscle tone.

Moreover, functionalized nanotubes, particularly those coated with extracellular matrix proteins such as laminin, promote neuronal adhesion and axonal extension, which may guide regenerating or stressed axons and help maintain synaptic alignment [115]. Additionally, the mechanical strength of CNT-based constructs may contribute to stabilization of the NMJ, reducing the likelihood of synaptic detachment and preserving motor unit integrity.

Experimental evidence suggests that CNTs can modulate intracellular calcium dynamics, which are essential for acetylcholine release and effective neuromuscular transmission [116]. Moreover, engineered nanotube platforms can be designed to deliver neurotrophic factors locally, providing targeted trophic support that enhances neuronal survival and function [117].

Importantly, such approaches are envisioned as adjunctive rather than replacement therapies. While current pharmacological treatments for SMA target the underlying genetic deficiency in SMN protein, nanotube-based interventions aim to reinforce the damaged neuromuscular infrastructure. By strengthening the electrical, structural, and biochemical aspects of the NMJ, this strategy seeks to preserve existing motor units and maximize functional outcomes.

### 6.3. Emerging Nanomaterial Platforms for Neuromuscular Junction Stabilization

While CNTs are the pioneers of this field, several other nanomaterials offer unique advantages that make them specifically suited for fixing the broken link between motor neurons and muscles (Table 2).

First, boron nitride nanotubes possess a game-changing property—piezoelectricity—meaning that they convert mechanical energy (like movement or ultrasound) into electrical signals [118]. In the NMJ, these nanotubes can be activated by external ultrasound to provide wireless electrical stimulation to the synaptic bulb, helping to maintain the signal transduction even if the axon is physically failing, without needing a battery or wired connection [119].

Second, silicon nanowires are semiconducting structures that can interface with cells with extreme precision and can generate a local electrical field, creating “optogenetic-like” interfaces where light is used to trigger the release of acetylcholine at the NMJ, bypassing the need for a healthy action potential to travel all the way from the spine [120].

Third, graphene and reduced graphene oxide in the NMJ are suggested to be able create meshes, wrapping around an entire muscle fiber bundle and acting as a conductive net that catches any stray signals from degenerating motor neurons, distributing them evenly across the muscle to prevent patchy atrophy [121].

Fourth, conductive polymer nanofibers, unlike the rigid carbon or silicon, have a mechanical stiffness very close to that of real nerves, and are excellent for long-term scaffolding where the goal is to have the neuron actually grow into the material [122].

Finally, self-assembling peptide nanotubes are entirely biological and made of amino acid sequences, designed to mimic the extracellular matrix. While they are not primarily used for conductivity but for biochemical signaling, these nanotubes can be programmed with the exact protein sequences that signal motor neurons to stay attached to a muscle [123,124].

The most advanced current research is moving toward hybrid systems. For example, a scaffold made of conductive polymers (for softness) reinforced with CNTs (for signal strength) and coated with peptide nanotubes (for biological signaling) [125,126,127]. By using this synergistic approach, it is possible to address the electrical, mechanical, and chemical needs of the NMJ simultaneously.

## 7. Neuromuscular Junction Instability in Spinal Muscular Atrophy as a Target for Localized Structural Augmentation

The rationale for this section follows directly from the clinical and electrophysiological observations summarized in Section 2. In treated SMA, persistent neuromuscular transmission defects, fatigability, reduced endurance, and a limited residual motor unit substrate indicate that the relevant therapeutic problem is not only weakness per se, but instability of distal motor unit performance under repeated activation. Section 7 therefore translates these residual abnormalities into a physiologically interpretable framework that defines how the potential usefulness of CNT-based adjunctive interventions may be assessed. The primary clinical issue with neuromuscular junction dysfunction in SMA patients treated with gene therapy is consistent reliable activation of all motor units (output) in a muscle, particularly since the muscle has a limited reserve within which to function. The relevant clinical question is not only how much force can be produced by a muscle on one occasion, but rather how well neuromuscular transmission allows for continual effective use of a muscle at repeated intervals (time) based upon the combined activation of all motor units [128].

The reliability framework defines an important practical aspect of untreated SMA, i.e., that functional limitations present themselves through task decrements, day-to-day variability, and disproportionate fatigue relative to peak performance [129]. In this context, NMJ represents a feasible therapeutic interface due to its anatomical localization and decisive physiology. Consequently, a NMJ stabilization strategy can only be meaningfully defined by its effects and measurements at the NMJ interface, as opposed to broadly restating upstream disease biology [23].

To make NMJ instability actionable as a therapeutic target, it should be operationalized as a phenotype with explicit, measurable dimensions. A practical definition encompasses three common aspects: transmission reliability, motor unit reserve/continuity, and clinically significant functional reserve. Transmission reliability refers to the probability that a recruited motor unit will maintain effective neuromuscular transmission during repeated activation, rather than undergoing activity-dependent failure of transmission. Operationally, it reflects the local safety margin of neuromuscular transmission and may be inferred from the electrophysiological evidence of decrement, jitter, blocking, or other signs of impaired junctional stability. In this context, activity-dependent dropout denotes intermittent loss of effective transmission from motor units that are anatomically present and centrally recruited but fail to maintain consistent output during repeated activation. Motor unit reserve/continuity refers to the amount and integrity of residual motor unit substrate available to sustain stable output over time. In the present framework, this construct encompasses not only the number of surviving motor units, but also the consequences of collateral remodeling, limited compensatory buffer capacity, and the extent to which the remaining substrate is sufficient for local stabilization to translate into measurable functional benefit. Functional reserve refers to the clinically observable capacity to preserve task performance over time despite repeated activation demands. This is expressed through endurance, resistance to fatigability, and reduced performance decrement during daily activities or repeated motor tasks, rather than through maximal strength alone [130].

Functional reserve reflects how these constraints affect the patient in terms of declining performance over time and reduced endurance for daily tasks [131]. This composite definition provides a measure to differentiate between generic weakness and NMJ instability; a person may experience a consistently weak strength output but relatively consistent strength off the NMJ, or an individual who has a small amount of weak strength yet possesses high NMJ instability (e.g., poor performance under heavy loads) [132]. In this manner, by focusing on NMJ instability, it is a way to improve the second case.

A framework of a measurement hierarchy based on stability-first phenotypical characterizations is fundamental in understanding physiological interpretability. Within this framework, output stability denotes reduced repetition-to-repetition variability in neuromuscular performance, indicating that recruited motor units can sustain more consistent force generation across time. The greatest degree of mechanistic insight will derive from the greatest amount of physiologically relevant signals (i.e., signals closest to junctional physiology) and the least amount of confounding due to musculoskeletal adaptations. Therefore, the initial indicators of whether an intervention increases the effective safety margin (the ability to avoid injury) will be indicators of transmission reliability [133,134]. The second layer of the hierarchy consists of measures of motor unit continuity and motor unit longitudinal stability; these, with the first layer, will separate out true stabilization (motor unit substrate) from transient fluctuations or compensatory activity. Functional reserve outcome measures reflect clinically meaningful information but require concurrent interpretation with objective stabilization indicators due to orthopedic constraints, respiratory demands, training state, and compensatory strategy of the individual [135]. This hierarchy is not merely methodologically based but rather reflects a causal assumption: increased reliability of transmitted communication results in decreased activity-related loss of participants, and decreased loss of participants results in increased endurance and consistency.

A candidate selection framework will provide a basis for avoiding dilution of the target population and preserving the interpretability of the results. The minimum requirement to stratify descriptors is that there is evidence of instability that is appropriate/related. Instability may be inferred when objective measures are obtained that provide evidence of impaired reliability of transmission; or when there are limited motor unit reserves present; or when either clinically significant fatigue or clinically significant limitations in endurance have occurred. Stratification of descriptors will provide a more refined expected responsiveness to treatment, and will assist in differentiating between instability driven by NMJ factors and instability predominantly caused by non-NMJ factors [128,136].

Useful descriptors include timing of treatment relative to symptom onset, disease phase (developmental versus chronic), and the dominant performance limiter (fatigability-dominant versus fixed weakness). This structure also introduces an implicit ceiling concept: when reserve is extremely low, there may be insufficient substrate for stabilization to translate into meaningful functional change; conversely, when instability is minimal, detection of benefit becomes difficult [137].

Within this conceptual space, localized structural augmentation can be defined as a distinct therapeutic category: an intervention aimed at increasing the mechanical cohesion and functional reliability of the NMJ interface under repeated activation [138]. “Structural” here does not mean rigid scaffolding alone; it denotes the preservation of micro-architecture and maintenance of effective presynaptic–postsynaptic apposition that reduces susceptibility to activity-dependent failure. “Localized” emphasizes mechanistic interpretability. A genuine junctional effect should be strongest in targeted muscle groups and should map onto measurable improvements in transmission reliability before it manifests as broader functional gains [139]. The most realistic clinical benefit profile of this category is improved stability and endurance rather than dramatic increases in maximal strength because raising the safety margin primarily reduces dropouts and output variability [140]. The value of this framing is that it sets a clear physiological chain from target to outcome and defines success in terms that are aligned with the dominant limitation experienced by many treated individuals.

Within this framework, the usefulness of a CNT-based adjunctive intervention is not inferred from maximal strength gain alone. Rather, it is assessed according to whether the intervention produces a logically ordered pattern of benefit: first, improved transmission reliability at the neuromuscular interface; second, greater output stability of the residual motor unit pool; and third, clinically meaningful preservation of functional reserve, expressed primarily as improved endurance and reduced fatigability. This sequence is important because it distinguishes true junctional stabilization from nonspecific behavioral adaptation, training effects, or compensatory motor strategies.

## 8. A Hypothesis-Driven Conceptual Model of Nanotube-Assisted Motor Unit Stabilization in Treated Spinal Muscular Atrophy

The originality of the present review lies not only in considering CNT-based interfaces as adjunctive candidates, but in embedding them within a falsifiable conceptual model in which improvement in junctional reliability is expected to precede, and mechanistically support, subsequent gains in motor unit output stability and functional reserve. The present conceptual model proposes nanotube-assisted stabilization as an adjunctive strategy for treated SMA, intended to complement currently approved SMN-restoring therapies rather than replace upstream molecular correction. Its central physiological construct is motor unit output stability, defined here as the capacity of surviving, remodeled motor units to translate neural drive into consistent and sustainable neuromuscular output during repeated activation over time. The conceptual model does not assume regeneration of lost motor neurons, reconstruction of entire motor circuits, or reversal of established denervation. Instead, it hypothesizes that in a system with limited remaining motor unit reserve, improving the reliability of the distal motor unit output may reduce activity-dependent failure and extend functional reserve even without restoring the original motor unit pool.

In this framework, “adjunctive” refers specifically to combination with existing SMN-restoring therapeutic strategies, including antisense oligonucleotide-based splicing correction, gene replacement, and small-molecule splicing modifiers. The proposed nanotube-assisted interface is therefore not conceived as a competing disease-modifying therapy, but as a downstream support strategy intended to address persistent motor unit and neuromuscular junction instability that may remain despite successful SMN augmentation. This distinction is central to the conceptual model: the target is not the primary genetic defect itself, but the residual physiological vulnerability of the distal motor unit in treated disease.

Within the present conceptual model, “localized” does not imply systemic neural rescue, but anatomically restricted action at or near the distal motor unit and neuromuscular junction interface of selected target muscles. The rationale for such localization is mechanistic interpretability: if the intervention acts at the neuromuscular interface, the earliest detectable signal should emerge in the targeted musculature as improved transmission reliability, reduced activity-dependent dropout, and greater output consistency before broader functional changes become evident. Thus, localization in this context refers to site-specific physiological action and measurement, rather than a claim of whole-system restoration.

Importantly, the conceptual model does not propose that nanotube-assisted interfaces alter the central recruitment order of motor units. Recruitment remains determined by the physiological organization of motor control. Rather, the proposed intervention is hypothesized to act downstream of recruitment, at the level of the distal motor unit output interface, by improving the likelihood that an already recruited motor unit will maintain effective neuromuscular transmission during repeated activation. In other words, the expected benefit is not recruitment of previously unavailable motor units through altered central drive, but greater stability, continuity, and reliability of output from motor units that are still anatomically present but functionally vulnerable.

**Figure 2:**
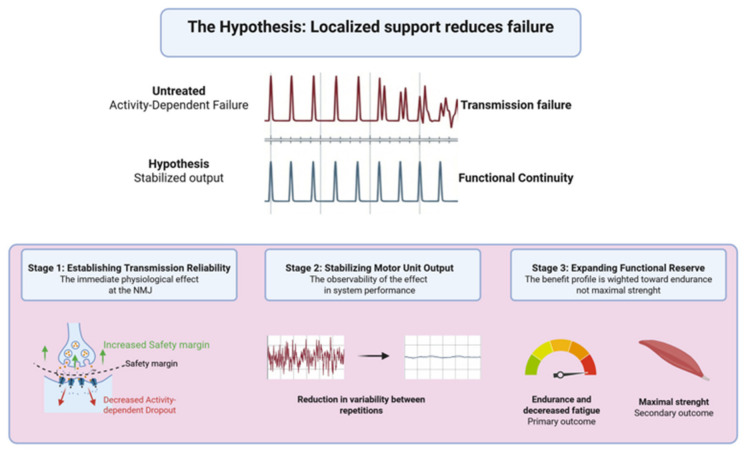
From transmission failure to functional continuity: A conceptual framework. Created in BioRender. Fajkić, A. (2026) https://biorender.com/nlmw6hz.

The core hypothesis of the conceptual model is that a localized nanotube-enabled interface, implemented at or near the distal motor unit and neuromuscular junction, can increase the motor unit output stability by reducing the probability of activity-dependent neuromuscular transmission failure and by preserving functional continuity at the neuromuscular interface [141]. In this context, functional continuity refers to the capacity of surviving, already recruited motor units to maintain reliable and repeatable neuromuscular output during repeated activation, despite a limited and remodeled residual motor unit substrate. The hypothesis is deliberately falsifiable through an expected ordering of effects. If the intervention truly stabilizes the motor unit output, objective measures that directly reflect junctional reliability should improve before, or at least in parallel with, improvements in endurance-based function (Figure 2). A functional improvement in the absence of any objective signal of increased transmission reliability would weaken a stabilization interpretation and would instead suggest alternative explanations such as behavioral adaptation, training effects, compensation, or measurement variance [142]. Here, improved junctional reliability refers to a higher safety margin of neuromuscular transmission and reduced activity-dependent dropout, whereas consistent motor unit output refers to reduced variability in the capacity of already recruited motor units to sustain effective neuromuscular performance across repeated activation.

Physiological assessment within this conceptual model should therefore focus on measures that distinguish transmission reliability from broader functional adaptation. Candidate tools include repetitive nerve stimulation and single-fiber electromyography for the assessment of neuromuscular transmission instability, as well as motor unit-oriented techniques such as motor unit number estimation, motor unit number index, and high-density surface electromyography to characterize residual motor unit substrate, output variability, and longitudinal change. In this framework, improvement would be expected to appear first as greater transmission reliability and more stable motor unit output in targeted muscles, rather than as an immediate increase in maximal strength alone. These measures are also relevant for identifying the subgroup of patients with preserved-but-vulnerable residual motor unit substrate, in whom local stabilization would remain physiologically meaningful.

Within this conceptual model, the available CNT evidence from SCIs and related experimental systems should be interpreted as translational support rather than as direct clinical evidence for SMA. Its relevance lies in demonstrating that nanotube-enabled materials can function as localized structural and bioelectrical interfaces in compromised motor systems, thereby providing mechanistic plausibility for an adjunctive SMA-focused strategy. Accordingly, the present framework does not claim established clinical efficacy of CNTs in SMA but proposes a testable rationale for their future evaluation as localized supportive interventions in treated disease.

The upper schematic compares untreated activity-dependent transmission failure, shown as irregular and intermittently dropping output during repeated activation, with the hypothesized post-intervention state of stabilized output. Here, functional continuity denotes the preservation of sufficiently reliable neuromuscular transmission to sustain reproducible output from surviving, already recruited motor units across repeated activation despite limited residual reserve. The term does not imply motor neuron replacement, reconstruction of the original motor unit pool, or altered central recruitment, but rather the reduction in distal output failure at or near the neuromuscular junction. The lower schematic depicts the proposed temporal sequence of effects. Stage 1 shows the immediate local junctional effect, defined by increased safety margin and reduced activity-dependent dropout. Stage 2 shows stabilization of motor unit output, reflected by reduced variability between repetitions and greater consistency of performance over time. Stage 3 shows the predicted functional consequence, namely expansion of the functional reserve, expected to manifest primarily as improved endurance and reduced fatigability, with maximal strength represented as a secondary outcome. The figure therefore summarizes a falsifiable adjunctive conceptual model in which improvement in transmission reliability is expected to precede, or at least accompany, functional gains.

The intervention described is based on functional requirements, and not determined by type of nanotube, type of coating, or method of fabrication to facilitate generalizability and to avoid a platform specific materials narrative. In mechanistic terms, the effect must be localized, such that the physiological signal will be clearest at the site(s) of interest (i.e., the targets) and there will be a spatial pattern (site-specificity) supporting causality (the relationship between the pathological event and local muscle action). The effect of transmission requires load to manifest. The pathological event of interest is not the baseline excitability (rest) of muscle, but the probability of transmission being below threshold during repetitive activation. It is necessary to implement this approach in a manner that adheres to the mechanisms of chronic remodeling. Therefore, the model allows for the assumption that any motor units affected by the interventive muscle actions will have experienced a size increase and to be functionally weak. Therefore, an effective interface must be able operate in a substrate that has been remodeled and will not require an acute rescue environment. Finally, any intervention must also retain the status of an adjunct to postural activities, which means that it is compatible with continuing systemic therapeutic activities and serves as a source of additional support rather than as a substitute for upstream correction.

In this conceptual model, the progression through the causal chain occurs in three stages. During the first stage, there is an increase in the effectiveness of transmission reliability locally (as demonstrated by a decrease in activity-dependent dropout and an increase in the junctional safety margin, within the specific motor unit population). During the second stage, the motor unit output stabilizes (as evidenced by a reduction in variability between repetitions and improvement in consistency of the neuromuscular performance of an individual over time). Finally, during the third stage, the functional reserve expands and is most likely represented by improved endurance and decreased fatigability during activities that previously resulted in rapid decrements across all repetitions. The chain suggests a benefit profile that is mainly weighted toward stability and endurance, with a smaller or slower development of maximal strength. This differential development pattern is expected because increasing the reliability of the transmission of information decreases the number of intermittent failures, as well as increasing the peak force generation. In a system with limited reserves, reducing intermittent failure may be tremendously important.

The conceptual model further predicts phenotype-dependent responsiveness and introduces a practical “sweet spot” concept for patient selection. The greatest benefit is expected in patients who show demonstrable junctional instability together with preserved-but-limited residual motor unit integrity, meaning that sufficient substrate remains for stabilization to translate into measurable functional gain while instability is still prominent enough for improvement to be detected. In contrast, when the motor unit reserve is extremely depleted, functional leverage may be too limited for local stabilization to yield clinically meaningful benefit. Conversely, when instability is minimal, effect sizes may be small and difficult to attribute mechanistically. In this sense, residual motor unit integrity functions not only as an outcome-related construct, but also as a selection axis that helps define which treated SMA patients are most likely to respond to CNT-based adjunctive strategies.

It is possible to express minimal model-consistent performance measures independently of those derived from clinical trial design features. Supportive adverse findings can exist independently of measurable improvements in objective parameters of transmission reliability within the targeted musculature, as well as independent of other objective performance metrics (rehabilitation, performance) and demonstrate success in several dimensions relative to specific applications (technical specification, anatomical specificity). In addition, the time sequence will compare changes in objective transmission reliability to changes in functional reserve or stability for each individual while using the definitions used in many research studies. These outputs define what the model means by stabilization and set the boundary between mechanistic plausibility and descriptive association. They also clarify what the model does not claim: it does not assume motor neuron replacement, broad synaptogenesis, or reversal of established neuron loss; it assumes that reducing distal output failure can meaningfully extend functional reserve in a remodeled motor unit system.

Focusing on the motor unit stability as an explicit physiological construct will allow this complementary model to provide a transparent link between the intervention class and measurable outcomes while still maintaining falsifiability. The model uses objective measures of reliability as the organizing principle, is based on site-specificity, and has a clinically relevant endurance-weighted efficacy profile for patients with SMA. This structure also creates a disciplined basis for translational development but is conceptually distinct from both the mechanics of the platforms as well as from later specification of clinical pathways, prediction testing and future directions of the development process. Clinical translation of CNT-based adjunctive therapy for SMA must address key safety and feasibility considerations, including biocompatibility and long-term CNT persistence, mechanical matching to neural tissue compliance, controlled electrical properties to prevent aberrant excitability, and scalable, standardized manufacturing for clinical-grade applications [97,98,99]. Ongoing large-animal studies and advances in nanotube surface functionalization are progressively addressing these barriers, and the integration of CNT interfaces with established neuromodulation modalities—including closed-loop stimulation and optogenetic approaches—represents a promising direction for future clinical development [100,101,102]. Together, these considerations define a translational roadmap from preclinical SCI and ALS models to first-in-human applications in SMA adjunctive therapy.

## 9. Safety, Biocompatibility, and Ethical Constraints in Nanotube-Based Neurological Interventions

Nanotube-based interventions hold transformative potential for stabilizing motor neurons and NMJs in SMA, yet their clinical translation demands exhaustive scrutiny of the safety, biocompatibility, and ethical constraints. These nanomaterials, particularly CNTs, must navigate unique challenges posed by their nanoscale properties in the delicate CNS environment. This section delineates these considerations, drawing from preclinical data, regulatory frameworks, and bioethics discourse to inform adjunctive therapy development for SMA, ALS, neurodegenerative diseases, and SCI [143].

Safety evaluation of CNTs in neurological applications prioritizes mitigating nanomaterial-specific hazards such as cytotoxicity, genotoxicity, and systemic biodistribution. Pristine CNTs, due to their asbestos-like fibrous morphology and surface reactivity, can trigger oxidative stress via ROS overproduction, leading to lipid peroxidation and apoptosis in motor neurons—pathologies mirroring SMA denervation cascades. Functionalized CNTs, conjugated with PEG or biocompatible polymers, exhibit markedly reduced toxicity; for instance, PEGylated MWCNTs in SCI rodent models showed <2% neuronal loss at therapeutic doses (5–20 mg/kg), with half-lives under 30 days via renal clearance [88,143,144,145].

Dose–response studies underscore thresholds: acute exposures below 10 μg/mL maintain >95% motor neuron viability in iPSC-derived NMJ organoids, but chronic dosing risks microglial priming and neuroinflammation. Hemocompatibility assessments per ISO 10993-4 reveal minimal platelet aggregation for carboxyl-modified single-walled CNTs (SWCNTs), critical for intrathecal delivery bypassing the BSCB. Long-term risks include bioaccumulation in lysosomes, potentially amplifying alpha-motor neuron vulnerability in SMA type I patients; MRI-tracked CNTs in non-human primates confirm clearance within 6–12 months, averting granulomatous responses [88,145,146,147,148]. Regulatory safety mandates encompass ICH S9 guidelines for neural therapeutics, integrating in silico modeling (e.g., QSAR for ROS prediction) with GLP-compliant assays. Post-implantation monitoring via telemetry-embedded CNTs could enable real-time pharmacovigilance, addressing gaps in SCI trials where 15–20% adverse events stem from nanomaterial aggregation [88].

Furthermore, biocompatibility hinges on seamless CNT integration with NMJ architecture, preserving synaptic vesicle release and acetylcholine receptor clustering. SWCNTs, with diameters approximating axonal microtubules (1–2 nm), facilitate scaffold-guided neurite outgrowth, boosting NMJ maturation by 40% in SMA patient-derived cultures. Hydrogel–CNT composites mimic extracellular matrix stiffness (1–10 kPa), promoting agrin-mediated postsynaptic differentiation without ectopic synaptogenesis [145,147,148]. Immune biocompatibility challenges arise from pattern recognition receptor activation; pristine CNTs elicit NLRP3 inflammasome signaling, elevating IL-1β in astrocytes and hastening muscle atrophy. Biomimetic functionalization—e.g., laminin-111 or RGD peptides—suppresses this by 80%, fostering M2 macrophage polarization and Schwann cell remyelination in demyelinated SCI models. In vivo, CNT–chitosan scaffolds in rat hemisection SCI restore NMJ electrophysiology (CMAP amplitudes >70% baseline) with fibrosis limited to 5–8% scar volume [88,145,147]. Barrier biocompatibility is optimized via zwitterionic coatings achieving zeta potentials of −25 mV, enabling BSCB traversal with <1% parenchymal leakage. Longitudinal biocompatibility in large-animal NMJ assays (e.g., porcine SMA analogs) reports 85–90% synaptic stability at 3 months, rivaling viral vectors but without immunogenicity. Advanced metrics such as multi-electrode array (MEA) recordings validate preserved NMJ fidelity, essential for SMA adjunctive use alongside nusinersen [88,145,148].

A subject worth highlighting is that ethical oversight in nanotube neurological interventions upholds Beauchamp and Childress’ four principles, with UNESCO’s neurotechnology ethics emphasizing human dignity amid enhancement risks. Informed consent protocols must detail probabilistic harms (e.g., 5–10% infection risk from implantation) and unknowns such as intergenerational CNT persistence, particularly for pediatric SMA cohorts where assent involves developmental psychologists. Vulnerability in neurodegenerative trials necessitates stratified recruitment to avert coercion, aligning with CIOMS guidelines for justice in resource-limited settings [146,149,150]. Privacy imperatives address CNTs’ electropotential for neural recording, invoking “neurorights” against unauthorized decoding of motor intent. BCI ethics precedents mandate opt-out clauses and federated learning for data minimization. Dual-use dilemmas—therapeutic CNTs adapted for cognitive augmentation—demand anticipatory governance, as per the Nuffield Council recommendations for horizon scanning [143,149,150,151]. Importantly, equity constraints highlight access disparities; nanotube therapies, projected at $75,000–$150,000/course, risk exacerbating global SMA burdens without tiered pricing or WHO essential medicine status. Inclusive ethics boards, incorporating patient advocates, ensure culturally sensitive trial designs [144,150].

Regarding the regulatory pathways in this setting, FDA and EMA classify CNT-neural hybrids as combination products under 21 CFR 3.0, requiring cGMP scaling and nanomedicine-specific CMC dossiers. Characterization mandates (TEM, Raman spectroscopy) address polydispersity impacting NMJ targeting. IND-enabling studies integrate ADME profiling, with CNTs’ prolonged t1/2 (weeks) necessitating pediatric extrapolations per the FDA’s SPeDAc framework [88,143,145,146]. Translational barriers include interspecies NMJ scaling; murine 90% stabilization efficacy wanes to 60% in ovine models due to gyrified cords. Phase I trials prioritize safety run-ins with surrogate NMJ biomarkers (e.g., serum NCAM). Post-approval, REMS programs monitor NMJ function via wearable EMG, bridging SCI precedents [147,148,150].

Finally, safety advances leverage machine learning for toxico-genomics, predicting CNT–neuron interactions with 92% precision. Biocompatibility evolves through 4D bioprinted NMJ-on-chips for high-throughput screening. Ethically, decentralized trials with blockchain consent enhance trust [143,147,149]. Interdisciplinary consortia could halve development timelines, projecting 25–40% NMJ preservation in SMA by 2030, complementing gene therapies [144,145].

## 10. Translational and Clinical Considerations, Testable Predictions, and Future Directions for Nanotube-Assisted Adjunctive Therapy in Spinal Muscular Atrophy

Translating nanotube-assisted adjunctive therapy for SMA into the clinic requires a rigorous framework that links the mechanistic stabilization of distal motor units to objectively measurable functional outcomes. The model’s temporal cascade predicts that early improvements in junctional reliability and reduced activity-dependent failure should precede gains in repetitive motor unit output and functional reserve, providing testable endpoints through electrophysiological assessments, wearable neuromuscular sensors, and endurance-based performance metrics. Patient selection based on residual motor unit integrity and phenotype-specific vulnerability may optimize therapeutic efficacy, identifying individuals most likely to benefit from adjunctive interventions.

Preclinical insights from ALS, SCI, and other neuromuscular disorders inform interface design, dosing strategies, and safety monitoring, while regulatory pathways must address long-term biocompatibility, clearance, and potential off-target effects of carbon nanotubes. Ethical considerations, including informed consent in vulnerable pediatric populations and equitable access, remain paramount. Early-phase studies should prioritize mechanistically relevant biomarkers that correlate with clinically meaningful functional gains, enabling iterative refinement of the intervention before broader implementation.

Future research should explore comparative efficacy alongside existing SMN-restoring therapies, optimize interface parameters to maximize junctional stabilization, and evaluate extrapolation to related motor neuropathies. Integration of biomimetic functionalization, high-throughput NMJ-on-chip screening, and predictive modeling can accelerate translation while mitigating patient risk.

Ultimately, nanotube-enabled interventions offer a mechanistically informed adjunct to current SMA therapies, aligning physiological plausibility with functional outcomes. By bridging objective motor unit stabilization, measurable performance gains, and patient-centered endpoints, this approach establishes a structured translational pipeline from preclinical validation to clinical application, representing a transformative step toward extending endurance, improving functional reserve, and enhancing quality of life for individuals with SMA and related neuromuscular disorders.

## Figures and Tables

**Table 1:** Comparative features of SMA, ALS, and spinal cord injury relevant to nanotube-assisted motor unit stabilization.

Feature	SMA	ALS	SCI
**Primary cause**	Biallelic SMN1 deletion/mutation → SMN protein deficiency	TDP-43, FUS, SOD1 aggregation; RNA metabolism dysfunction; sporadic or familial	Mechanical trauma → primary axonal transection + secondary excitotoxic/inflammatory cascade
**Motor neuron type affected**	Lower motor neurons (spinal alpha motor neurons); proximal > distal	Upper and lower motor neurons; fast-twitch motor units selectively vulnerable	Motor neurons below lesion level; upper motor neuron tracts disrupted
**NMJ pathology**	Presynaptic terminal swelling/retraction; fragmented AChR clusters; reduced vesicle release; incomplete denervation	Presynaptic alterations precede motor neuron death; synaptic vesicle machinery disrupted; dying-back degeneration	NMJ instability via anterograde (loss of descending input) and retrograde (denervated muscle) signaling; muscle atrophy
**Progression pattern**	Chronic, developmental onset; partially arrested by SMN-restoring therapies; residual NMJ instability persists	Progressive and irreversible; excitability dysregulation → hyperexcitability → hypoexcitability → death	Acute primary injury followed by chronic secondary degeneration; partial spontaneous recovery possible
**Current standard therapies**	Nusinersen, onasemnogene abeparvovec, risdiplam (SMN restoration); no therapy targeting NMJ directly	Riluzole, edaravone (symptomatic); no disease-modifying therapy reverses neurodegeneration	Surgical decompression, rehabilitation; neuroprotective agents under investigation
**Unmet therapeutic need**	Residual motor unit vulnerability, fatigability, and NMJ instability despite SMN restoration	Synaptic stabilization prior to motor neuron loss; no effective anti-synaptopathy strategy	Axonal regeneration across lesion; NMJ reconnection; circuit remodeling support
**Rationale for CNT adjunctive intervention**	Structural and bioelectrical NMJ stabilization in surviving, remodeled motor units; trophic support of distal axon terminals; directly addresses residual post-SMN-restoration deficits	Preclinical CNT evidence supports MuSK signaling preservation and NMJ integrity in SOD1 models; informs CNT interface design for SMA	SCI provides rigorous in vivo translational platform; CNT scaffolds demonstrate axonal regrowth, synaptic density improvement, and circuit reorganization directly applicable to SMA

**Table 2:** Comparative analysis of nanomaterials for NMJ repair.

Nanomaterial	Primary Strength	Use in NMJ Stabilization	Biocompatibility
**Carbon Nanotubes (CNTs)**	High Conductivity & Strength	Creating a conductive bridge to bypass axonal degeneration and restore signal.	Requires functionalization to reduce toxicity.
**Boron Nitride (BNNTs)**	Piezoelectric Power	Wireless stimulation; converting external ultrasound/movement into electrical pulses for the synapse.	Highly stable; often more chemically inert than CNTs.
**Silicon Nanowires (SiNWs)**	Optoelectronic Interface	Using light-activated triggers to control neurotransmitter release at the synaptic bulb.	Excellent integration with cellular membranes; standard in bioelectronics.
**Graphene/rGO**	Surface Area & Mobility	Wrapping muscle fibers in a conductive net to ensure uniform contraction across the motor unit.	Can be manufactured as flexible “carpets” for tissue interface.
**Conductive Polymers**	Mechanical Matching	Long-term scaffolding that matches the “softness” of real nerve tissue, reducing inflammation.	Highly biocompatible; degrades slowly as natural tissue regrows.
**Peptide Nanotubes**	Bio-Mimicry	Delivering targeted biochemical cues to prevent synaptic detachment.	Highest biocompatibility; eventually breaks down into amino acids.

## Data Availability

No new data were created.
